# Research on High- and Low-Temperature Characteristics of Bitumen Blended with Waste Eggshell Powder

**DOI:** 10.3390/ma14082020

**Published:** 2021-04-17

**Authors:** Xuancang Wang, Guanyu Ji, Yi Zhang, Yuchen Guo, Jing Zhao

**Affiliations:** School of Highway, Chang’an University, Xi’an 710064, China; WXC2005@chd.edu.cn (X.W.); 2019021079@chd.edu.cn (Y.G.); 2019021005@chd.edu.cn (J.Z.)

**Keywords:** eggshell powder, microstructural, bitumen material, high-temperature characteristic, low-temperature characteristic

## Abstract

The sustainability of resources is presently a major global concern. Sustainable construction materials can be produced by applying biological waste to engineering. Eggshells, as biological waste, are usually dumped in landfills or discarded. This causes many environmental problems including malodor, noise pollution, and serious waste of resources. To solve these problems, this study combined eggshell waste with bitumen materials for bio-roads construction. This paper investigated the impact of biological waste eggshell powder on the high- and low-temperature characteristics of bitumen materials. Scanning electron microscopy (SEM) revealed the microstructure of eggshell powder. The interaction between eggshell powder and asphalt was analyzed using Fourier transform infrared spectroscopy (FT-IR). The high- and low-temperature characteristics were investigated using conventional performance tests, and dynamic shear rheometer (DSR) and bending beam rheometer (BBR) experiments. These results indicate that eggshell powder (1) has a rough and porous microstructure; (2) has no apparent chemical reaction with asphalt; and (3) improves the consistency, hardness, and high-temperature characteristics. However, it reduces the plastic deformation capacity of asphalt, and the low-temperature crack resistance of asphalt cannot be improved. The research demonstrated that the application of eggshell powder in asphalt is feasible and has long-term resource and environmental advantages.

## 1. Introduction

With the increase in population and the acceleration of industrialization and urbanization, abundant solid waste is being generated [1]. It is reported that 2 billion tonnes of municipal solid waste is generated annually all over the world, and by 2050, it is estimated that 3.4 billion tonnes of waste will be produced annually around the world [2]. About 62% of these solid wastes are not properly treated, which causes air, soil, and water pollution, as well as global warming, and has severe implications for the sustainable development of the society and economy. On the one hand, social and economic development will lead to the production of solid waste. On the other hand, economic development also needs a lot of energy [3]. For the sake of building a green and sustainable development condition, it is necessary to effectively manage waste, and to produce more energy to meet the growing demand [3,4,5]. Upcycling is an effective means for sustainable waste management [6]. At present, municipal waste has been widely used in building materials, and remarkable results have been achieved [7]. Applications of municipal waste in construction materials are shown in Table 1.

It is well known that asphalt materials are widely used in road engineering construction because of their various advantages. Asphalt is extracted from crude oil, which is a non-renewable resource. The apparent consumption of petroleum asphalt in China has been increasing in recent years. Recent statistics show that apparent consumption was 5402.2 million tons in China, which continued to maintain rapid growth [23]. The demand for asphalt on pavement will continue to increase. In recent years, petroleum resources have decreased. Furthermore, many researchers have attempted to identify sustainable and renewable materials to replace petroleum products or reduce the use of petroleum products through a series of methods [24]. Moreover, there is a substantial demand for substitutes. Biological resources have received increasing attention from researchers owing to their wide sources, high yield, and renewability. For example, waste bio-oil ameliorates the low-temperature characteristics of bitumen binders [24]. The fatigue property of bitumen can be ameliorated by waste engine oil [25]. The comprehensive mechanical performance of bitumen materials modified by lignin powder has also been improved [26].

In addition to modifiers such as waste bio-oil and lignin, biological shell wastes such as crayfish and oyster shells have also been applied to asphalt [27,28]. Among all kinds of biological shell wastes, eggshells have received extensive attention. It is anticipated that about 7 million tons of eggshell waste are produced annually all over the world [29]. Eggshells as biological waste are generally dumped in landfills or discarded. This causes many severe environmental problems including malodor, bugs, and noise pollution [30,31]. Simultaneously, the acceleration of urbanization and the increase in infrastructure demand have caused a scarcity of natural resources. This, in turn, has resulted in a reduction in available landfills, and it has severe implications for the sustainable development of the society and economy [1,32]. In this scenario, researchers are compelled to identify solutions that can reasonably utilize this environmentally friendly biological waste.

At present, a substantial amount of research is being conducted on eggshell applications, and there have been relatively important results in many aspects [33,34,35,36,37,38]. A novel composite nanomaterial, eggshell/Ag, can effectively promote the catalytic degradation of organics and has substantial application prospects in the resolution of problems encountered in microbial treatment of sewage [39]. A 3D-printed biological reactive device was developed based on the selective permeability of eggshell membranes [40]. Eggshells as additive or coating agents for electrode materials prevent electrodes from contacting the electrolyte directly [41]. Biochar prepared by directly mixing eggshells with mushroom residues can effectively adsorb phosphorus and is important for reclaiming elements from sewage [42]. The catalysts prepared with natural waste eggshells, dolomite, and talcum powder can be used for synthesizing glycerol carbonate [43]. In the field of construction engineering, scholars have developed new foamed concrete using eggshell powder and palm oil fuel ash as natural and sustainable auxiliary cementitious materials. [44]. Polymerization of industrial waste fly ash and eggshell powder can also provide a sustainable building material that can be used for constructing subgrade and pavement base [30]. Eggshell powder as an inexpensive and sustainable stabilizing material is important for the strength of clay [45]. Clay blocks with eggshell powder can replace concrete for shielding buildings [46]. There are many comprehensive applications of eggshell waste in various fields.

As a low-cost biological waste, eggshells have significant sustainable development benefits, and the upcycling of this material can protect the environment and convert waste to wealth. Moreover, advocating the effective utilization of eggshell waste in asphalt conforms to the development concept of green buildings and can benefit the construction and resource recovery industries [47]. The application of eggshell waste in asphalt has remarkable economic and environmental benefits.

This study aimed to provide a new concept for recycling biological waste eggshells in bio-roads construction. It creatively applied waste eggshells to asphalt materials. To study the properties of eggshell powder asphalt, three kinds of eggshell powder asphalt were prepared with the proportions of 5, 10, and 15% (mass percentage). The microstructure of eggshell powder and chemical characteristics of eggshell powder asphalt were evaluated based on scanning electron microscopy (SEM) and Fourier transform infrared spectroscopy (FT-IR). Meanwhile, some conventional characteristics, dynamic shear rheometer (DSR), and bending beam rheometer (BBR) tests were carried out to evaluate the high-temperature and low-temperature performance of eggshell powder bitumen. The test results can be used as a reference for the application of eggshells in construction.

## 2. Materials and Methods

### 2.1. Materials and Sample Preparation

The material used for the test was 70# bitumen produced in Karamay (Karamay, China). The requirements are indicated in Table 2.

**Figure 1:**
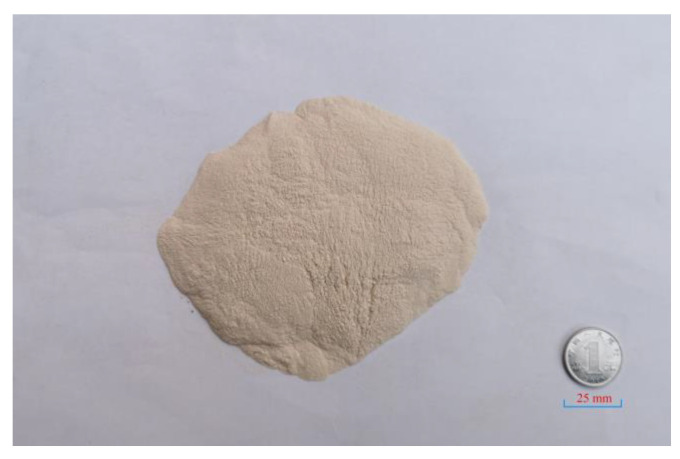
Biological waste: eggshell powder.

Eggshells (ES) were obtained from Anhui, China (Jiude health house). These eggshells were cleaned with clean water. Then, in an oven, eggshells were dried at 80 °C for 12 h. The eggshell waste was ground into powder in a grinder The particle size of the powder was approximately 0.125 mm, as shown in Figure 1.

A high-speed shear mixer rated at 300 W and manufactured by Shanghai Frank (Shanghai, China) was used in the sample preparation to maximize the mixing consistency. Based on previous study and test experience [19,49,50,51,52,53], the proportion of ES in the asphalt was 5, 10, and 15% (mass percentage), and the shear rate was optimized. The sample preparation process was as follows. First, the bitumen was put into an oven, and it was heated and melted at 135 °C. Next, the 5, 10, and 15% (mass percentage) of materials was weighed, and the corresponding content of biological waste eggshell powder was added gradually. Subsequently, the sample was stirred at 160–170 °C for 15 min at 1500 r/min, and premixing was performed to homogenize the material. Finally, biological eggshell powder asphalt was obtained by shearing at 3500 r/min for 40 min.

### 2.2. Microstructural Morphology

The microstructural morphology of the eggshell powder was observed by SEM to understand the morphology more intuitively and to explore its effect on the performance of bitumen materials. The test was carried out with the S-4800 instrument manufactured by Hitachi. The sample was sprayed with gold (Auari Chinese Medicine Machinery Co.Ltd, Wenling, China) before the test. The test results revealed the microstructural characteristics of biological eggshell powder.

### 2.3. Chemical Characterization

The FT-IR test adopted a TENSOR II Fourier transform infrared spectrometer produced by BRUKER. The experiment was conducted to investigate variations in functional groups before and after the addition of biological waste eggshell powder. The spectral range was 400–4000 cm^−1^.

### 2.4. Conventional Performance Testing of Asphalt

These experiments aimed to research the function of eggshell powder in the conventional properties of bitumen (SYD-2801F penetration, SYD-2806H softening point SYD-4508G ductility instruments, SHANGHAI CHANGJI GEOLOGICAL INSTRUMENT CO. LTD, shanghai, China). Based on the Chinese standard [54], the penetration (25 °C), softening point, and ductility (5 °C) of bitumen materials were investigated.

### 2.5. Rheological Characterization

**Figure 2:**
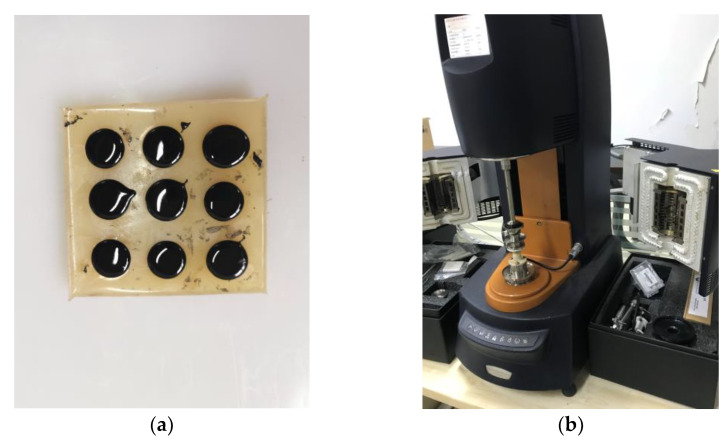
(**a**) (Dynamic shear rheometer) DSR and (Multiple Stress Creep Recovery) MSCR samples and (**b**) dynamic shear rheometer.

DSR tests are commonly used to indicate the viscoelastic characteristics of bitumen materials at various temperatures and frequencies [55]. In accordance with the Chinese standard [54] and United States standard (AASHTO T350-18) [56], the DSR and (multiple stress creep recovery) MSCR experiments were conducted by a dynamic shear rheometer (TA, USA), as indicated in Figure 2.

#### 2.5.1. Temperature Sweep

This research aimed to explore the rheological characteristics of bitumen materials at various temperatures. The experiment adopted the strain control mode and used a 25 mm smooth-surface metal plate with a parallel plate gap of 1 mm. The temperature range of this test was 40–70 °C, and the intervals were of 6 °C each. The asphalt materials were loaded at the rate of 10 rad/s. The complex modulus (|G*|) and phase angle (δ) of the samples were gained.

#### 2.5.2. Frequency Sweep

The temperature range of this test was 58–70 °C, and the intervals were of 6 °C each. The frequency sweep experiment was conducted under controlled strain conditions. Dynamic shear loads were applied to the bitumen material at loading frequencies from 0.1 to 100 rad/s. The |G*| and δ of the materials were gained to indicate the high-temperature rheological characteristics at different frequencies.

#### 2.5.3. Multiple Stress Creep Recovery

MSCR experiments were conducted at 64 °C by adopting unchangeable stresses (0.1 and 3.2 kPa). These stresses were loaded continuously for 1 s and then recovered for 9 s. This test involved 30 creep and recovery cycles (20 cycles at 0.1 kPa, followed by 10 cycles at 3.2 kPa). These first 10 cycles were for regulating the specimens. The cumulative time for completing the two-stage creep and recovery experiment was 300 s. The data of the final 10 cycles at 0.1 kPa and 10 cycles at 3.2 kPa were recorded. This first strain of each creep cycle was recorded as ε_0_. When this creep cycle was finished (1 s after the start of each cycle), the strain was recorded as ε_c_. The adjusted strain was obtained as ε_1_ = ε_c_ − ε_0_. The strain when this recovery cycle was finished (10 s after the start of each cycle) was recorded as ε_r_. The adjusted strain after the completion of the recovery period (10 s after the start of each cycle) was calculated by ε_10_ = ε_r_ − ε_0_.

The average recovery rate of the bitumen binder R and average irrecoverable creep flexibility J_nr_ were obtained from the data of the final 10 cycles at 0.1 kPa and these 10 cycles at 3.2 kPa. The calculation formula is as follows:

(1) $$R=\frac{\varepsilon_{1}-\varepsilon_{10}}{\varepsilon_{10}}$$

(2) $$J_{\mathrm{nr}}=\frac{\varepsilon_{10}}{\delta }$$

where δ is the stress exerted on the sample (0.1 or 3.2 kPa).

### 2.6. Bending Beam Rheometer Experiment

**Figure 3:**
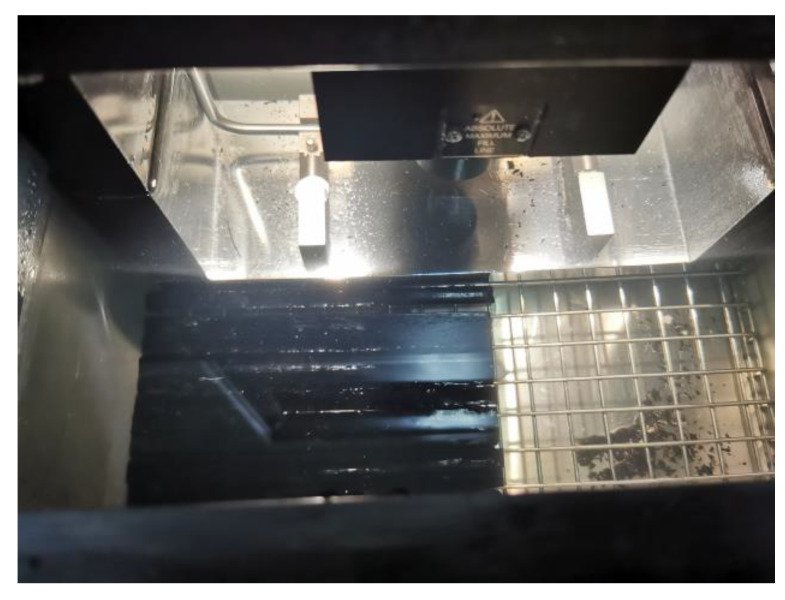
(Bending beam rheometer) BBR experiment.

Based on Chinese standards [54], the BBR experiment adopted a TE-BBR bending beam rheometer manufactured by Cannon, USA (Figure 3). The temperature range of this test was from −12 to 24 °C, and each interval was of 6 °C. The instrument was calibrated before the test. Then, the experiment was conducted based on pre-programmed procedures. The low-temperature characteristics were evaluated from the creep stiffness s and creep rate m at 60 s.

## 3. Results

### 3.1. Microstructural Morphology

**Figure 4:**
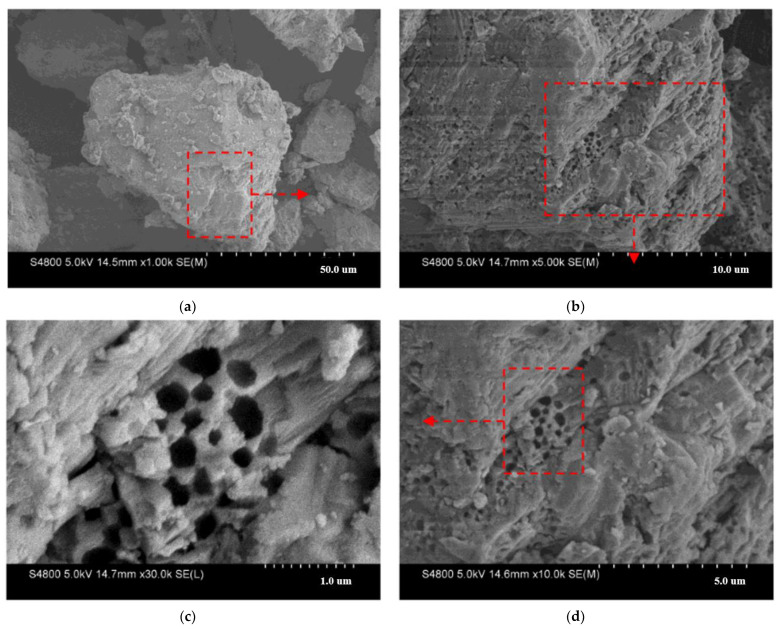
Microstructure image of eggshell powder: (**a**) 1000×, (**b**) 5000×, (**c**) 10,000×, (**d**) 30,000×. The part framed by red dotted lines represents observation range of particles at different multiples.

The microstructure of the eggshell powder was obtained by SEM. Figure 4 shows the SEM images at different magnifications. Figure 4a shows that the eggshell powder particles have a certain angularity. These particles were broken from larger particles. The smaller particles are attached to the larger particles, and the appearance of the structure is not smooth. Figure 4b–d shows that the particle elevation displays layered folds, has small holes that are of irregular sizes and are distributed non-uniformly for clarity and brevity, and has a higher microscopic roughness. This rough, porous structure of eggshells generally displays good adsorption properties [57]. This implies that biological eggshell powder can adsorb more asphalt, and the adhesion between eggshell powder and asphalt is better. Thereby, biological eggshell powder has an impact on the properties of asphalt materials.

### 3.2. Chemical Characterization

**Figure 5:**
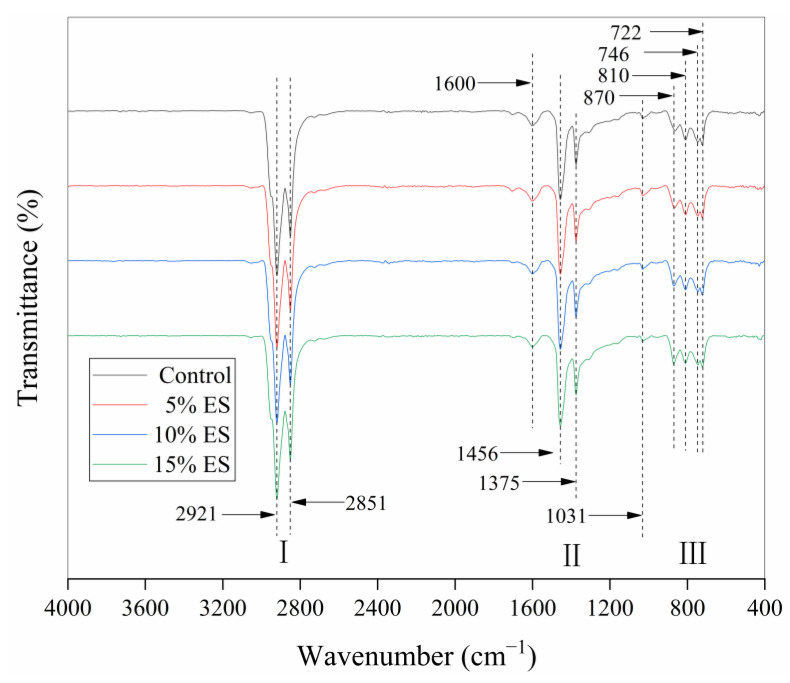
Infrared spectrum of asphalt binder.

The FT-IR test is a method that determines the chemical functional groups of materials. Chemical functional groups are atomic groups that have an impact on different reactions of the compound. The FT-IR experiment can characterize variations in the functional groups of asphalt materials [58]. Figure 5 shows the infrared spectra of asphalt materials. It shows that asphalt materials have three major absorption peaks in the infrared spectrum. In section I, asphalt has superior absorption peaks around 2921 and 2851 cm^−1^. In addition, the position of asphalt is identical to that of the control bitumen on the whole, which is generally the result of the C–H bond vibration of cycloalkanes and alkanes in asphalt materials. In section II, the second strongest peaks of the asphalt material are approximately 1456 and 1375 cm^−1^. The bending vibration of the C–H bond in asphalt material and the vibration peaks of CO_3_^−2^ introduced by eggshell powder are likely to be the main causes of the absorption peaks. The absorption peaks at 870, 810, 746, and 722 cm^−1^ in section III may be a result of the bending vibration of the C–H bond of aromatic compounds and the vibration peaks of CO_3_^−2^ introduced by eggshell powder.

According to the above analysis, the positions of the absorption peaks of the four groups are essentially identical, and there are no new absorption peaks. This phenomenon indicates that the interaction between eggshell powder and asphalt was mainly physical co-blending and that no new chemical groups were generated. Therefore, the eggshell powder did not affect the chemical structure of the bitumen materials.

### 3.3. Conventional Performance of Asphalt

**Figure 6:**
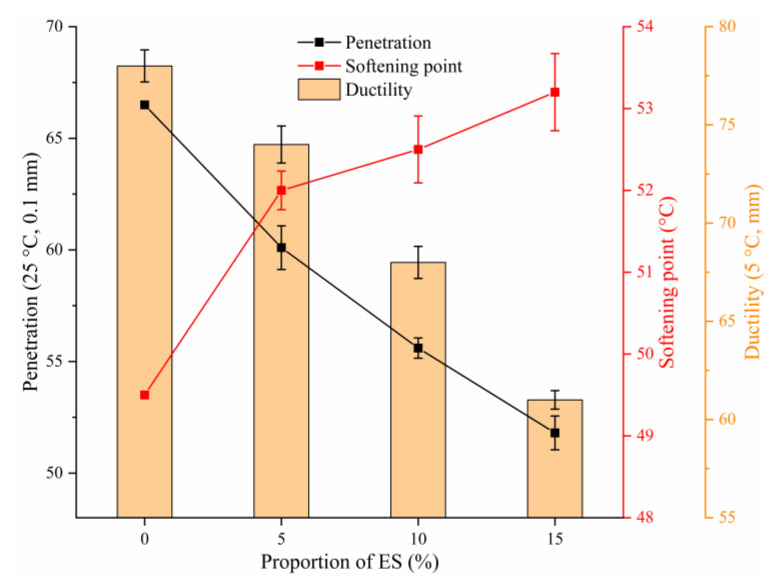
Conventional performance of asphalt results.

Experiments for determining the 25 °C penetration, softening point, and 5 °C ductility of bitumen binders were performed based on the Chinese standard [54]. In addition, the performance results of conventional bitumen containing 5, 10, and 15% eggshell powder were analyzed for a preliminary evaluation of the high- and low-temperature characteristics of bitumen materials. Figure 6 shows the conventional performance of asphalt results at different contents.

Penetration characterizes the viscosity and hardness of asphalt at a specified temperature. At present, it has a close relationship with the road performance of bituminous pavement. A larger penetration indicates a smaller thickness, lower hardness, and lower high-temperature performance [59]. The penetration of biological eggshell powder asphalt at 25 °C is revealed in Figure 6. In the figure, as the amount of eggshell powder rises, the penetration of bitumen material decreases, and asphalt gradually hardens. The results of the experiment tentatively showed that the biological eggshell powder could improve the consistency and hardness of asphalt and thereby its high-temperature properties.

Softening point is a crucial parameter that indicates the thermal stability of bitumen materials, which, in turn, reflects the rutting resistance of bitumen [60]. Figure 6 represents the variation in the softening point of bitumen with the content of eggshell powder. In the figure, as the content of eggshell powder rises, the softening point rises. The experiment further demonstrated that eggshell powder could increase the high-temperature property of bitumen materials.

The ductility index of asphalt indicates the capability of bitumen to withstand plastic deformation when it is stretched by an external tensile force. It is generally considered that the larger the ductility is, the higher the low-temperature deformation capacity, the higher the low-temperature crack resistance of pavements, and the less likely it is to crack [61]. In this study, the 5 °C ductility index was used to initially show the influence of biological eggshell powder on asphalt’s low-temperature property. Figure 6 shows the variation in the ductility of bitumen with eggshell powder content at 5 °C. In the figure, as the amount of eggshell powder rises, the ductility of bitumen decreases. The test results initially showed that the biological eggshell powder hardens bitumen and decreases its capability to undergo plastic deformation. This may be because the eggshell powder particles hinder the free movement of the bitumen mass. The higher the resistance capability is, the lower the ductility. However, it was observed that brittle failure occurred in the middle part of certain samples when the elongation was low and the diameter was large. Brittle damage became more apparent with an increase in the ES content. This may be because the presence of eggshell powder produces stress concentration and accelerates the failure of asphalt when the cross-sectional diameter of the asphalt specimen is directly stretched to a certain extent at a low temperature. Therefore, considering that the ductility index is related to many factors, the impact of eggshell powder on the low-temperature property requires further study.

### 3.4. Temperature Sweep

**Figure 7:**
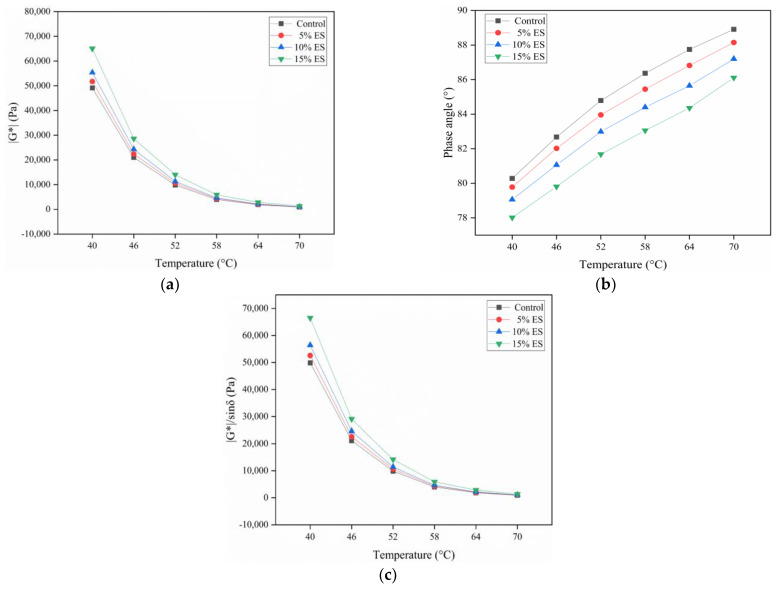
Temperature sweep results of 5, 10, and 15% eggshell powder bitumen: (**a**) complex shear modulus; (**b**) phase angle; (**c**) rutting factor.

The complex shear modulus |G*| is commonly used to indicate bitumen’s resistance to deformation under dynamic shear load [49,62]. Figure 7a represents the relationship between |G*| and temperature. Here, as the temperature increases, |G*| appears to decrease gradually. The reason is that the elastic component is dominant in bitumen when the temperature is low. This imparts bitumen with a higher capability to resist shear deformation, and |G*| is larger. With a gradual increase in temperature, asphalt gradually exhibits viscous properties. This results in a smaller |G*|. From another perspective, at an identical temperature, |G*| increases as the eggshell powder content increases. Within the temperature range, the growth rate of |G*| is the largest relative to the base bitumen at 15% ES content. This may be because with the addition of biological eggshell powder, the eggshell powder gradually adsorbed more asphalt, and the effective asphalt increased. This would have altered the asphalt structure, whereby the interaction between asphalt molecules would have become stronger than that in the matrix asphalt. Thus, the resistance to deformation increased, which resulted in an increase in |G*|. These experimental results showed that the biological eggshell powder significantly increased the resistance of bitumen to permanent deformation.

The phase angle δ characterizes the proportion of viscoelastic components in asphalt. In general, the larger δ is, the larger the proportion of viscous components, and the smaller the deformation recovery capacity [63]. Figure 7b indicates the relationship between δ and temperature. Here, the δ of the bitumen binders increases as the temperature increases. That is, with the temperature rises, the viscous component of biological eggshell powder bitumen rises and the proportion of unrecoverable deformation rises, while the resistance to high-temperature deformation declines. The reason may be that the thermal movement of asphalt molecules is accelerated with temperature rises. Therefore, as the temperature rises, asphalt becomes softer. At the same temperature, δ decreases as the ES content increases. Within the temperature range, the δ reaches a maximum extent of the reduction relative to the base bitumen at 15% ES content. The decrease of phase angle δ enhances the resilience of asphalt, on account of the eggshell powder changing the interaction of bitumen molecules. This reveals that ES could effectively reduce the viscous component in bitumen and improve the capability for deformation recovery.

|G*|/sinδ represents a property of pavements made using bitumen materials (namely, resistance to permanent deformation) and reflects the high-temperature property. In general, the larger |G*|/sinδ is, the smaller the deformation and the higher the rutting resistance [63]. Figure 7c indicates the variation in the rutting factor versus temperature. As shown in Figure 7c, |G*|/sinδ decreases as the temperature increases. |G*|/sinδ increases as the ES content increases at the same temperature. Within the temperature range, from that of the base bitumen, the |G*|/sinδ increased substantially at 15% ES content.

### 3.5. Frequency Sweep

**Figure 8:**
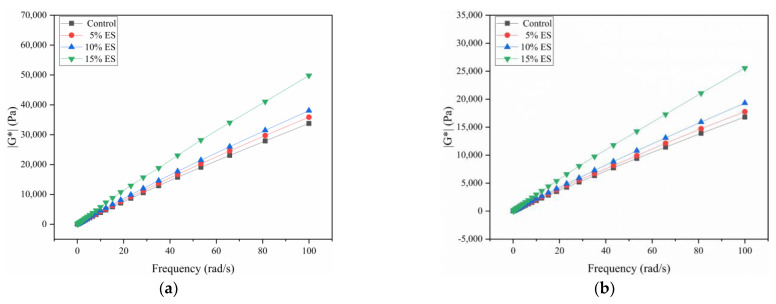

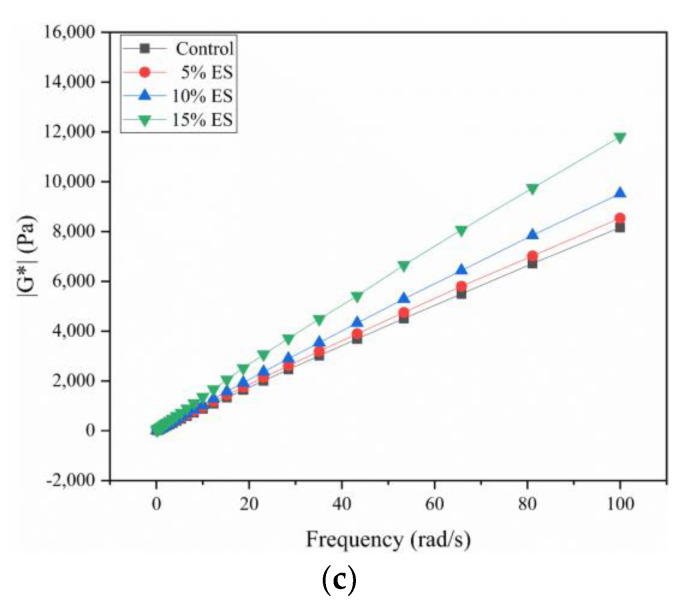
Frequency sweep results of 5, 10, and 15% eggshell powder bitumen: (**a**) 58 °C, (**b**) 64 °C, (**c**) 70 °C.

The mechanical response of viscoelastic materials is substantially influenced by the loading frequency. The smaller the frequency, the more apparent are its viscous characteristics. In addition, the larger the frequency is, the more apparent are its elastic characteristics. Frequency sweep tests can replicate pavement driving conditions [63,64]. Figure 8 represents the relationship between |G*| and the loading frequency at various temperatures. At the same temperature, |G*| increases as the loading frequency increases, and the elasticity is more apparent in the high-frequency region. It indicates that the high-temperature characteristics of bitumen materials increase as the loading frequency increases. This can be explained by the fact that as the frequency increases, the action time of the load on the bitumen binder decreases. Simultaneously, the shear deformation of the bitumen decreases, which shows that the complex modulus increases. Further analysis indicated that the |G*| of the bitumen bond increases with the addition of ES at the same temperature. Therefore, these test results further demonstrate that ES can enhance the high-temperature characteristics of bitumen materials.

### 3.6. Multiple Stress Creep Recovery

The MSCR experiment is performed to characterize viscoelastic response and resistance to permanent deformation by testing the recovery rate R and irrecoverable creep flexibility J_nr_ [65,66]. The MSCR test of biological eggshell powder asphalt at 64 °C was carried out based on the conclusions of conventional tests, and DSR temperature and frequency sweep experiments.

**Figure 9:**
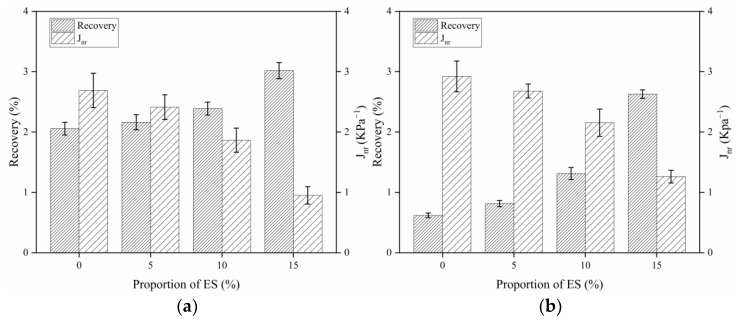
Average recovery rate R and irrecoverable creep flexibility J_nr_ of bitumen with different ES contents: (**a**) 0.1 kPa, (**b**) 3.2 kPa.

The average recovery rate R and average irrecoverable creep flexibility J_nr_ with various ES contents were obtained by MSCR tests. These are shown in Figure 9. The elastic component of bitumen material is commonly characterized using the R value: the larger the R value, the higher is the bitumen materials’ resilience. J_nr_ indicates the irrecoverable creep flexibility of a bitumen material at high temperatures: the smaller J_nr_ is, the higher is the resistance to permanent deformation. In Figure 9, the recovery rate of bitumen R (0.1 kPa) > R (3.2 kPa) and J_nr_ (0.1 kPa) < J_nr_ (3.2 kPa) at the same ES content. This could explain the phenomenon of deeper rutting with higher tire pressure in the actual pavement. Furthermore, the addition of ES increases R and decrease J_nr_ from those of the matrix bitumen. This indicates that the ES improved the rutting resistance at different stress levels to a certain extent. This is congruous with the experimental conclusions observed in the DSR temperature and frequency scanning. This improvement mechanism may be as follows: under the action of a mechanical force, the adsorbed layer of asphalt material becomes thinner, and the colloidal structure becomes looser. In addition, the incorporation and dispersion of ES can significantly limit the disruption of the colloidal composition, which can significantly increase the elasticity of the bitumen material.

### 3.7. Bending Beam Rheometer Experiment

**Figure 10:**
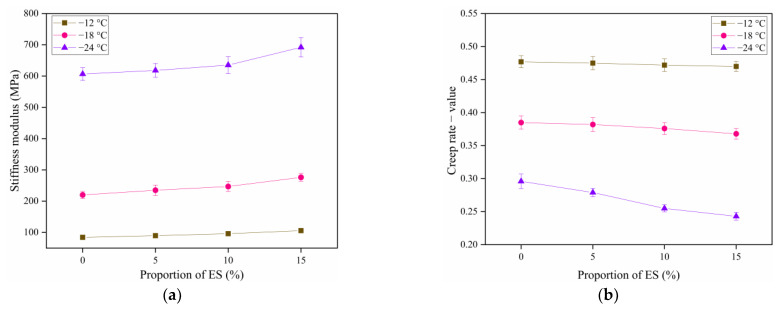
Stiffness modulus and creep rate of bitumen materials with different eggshell powder contents: (**a**) stiffness modulus (s); (**b**) creep rate (m − value).

The BBR experiment evaluates the creep degree of bitumen binders at lower temperatures and constant loads. The creep stiffness gained can efficiently explain the crack resistance of bitumen material at low temperatures [67]. The variation trends of the creep stiffness modulus s and creep rate m with ES content are represented in Figure 10. An important parameter, s reflects the bitumen materials’ low-temperature rheological characteristics and thereby indicates their resistance to deformation at low temperatures; while m indicates the rate of variation in the stiffness of the bitumen materials, thereby reflecting their stress relaxation characteristics. In general, smaller s and larger m values indicate better low-temperature characteristics of bitumen binders [68].

In Figure 10, as the temperature reduces, s increases and m decreases. This reveals a certain temperature sensitivity. The figure indicates that as the temperature decreases, the low-temperature deformation resistance and stress relaxation property of bitumen gradually decrease, whereas the hardness and brittleness of asphalt gradually increase.

At −12 and −18 °C, as the ES content rises, the s of bitumen increases, whereas its m decreases. However, the amplitudes of variation in s and m are not significant. The variations satisfy the requirements of 60 s asphalt s < 300 MPa and m − value > 0.3. This points out that at −12 and −18 °C, eggshell powder has a low impact on the low-temperature crack resistance of bitumen materials.

At −24 °C, s gradually increases as the ES content increases. Meanwhile, m exhibits the converse trend. The sensitivity of the anti-cracking characteristics of bitumen material at −24 °C to the content of eggshell powder is higher than that at −12 and −18 °C. Furthermore, it does not satisfy the requirements of 60 s asphalt s < 300 MPa and m − value > 0.3. This shows that at −24 °C, eggshell powder substantially reduce the low-temperature relaxation capacity of bitumen and increase the probability of cracking. Furthermore, asphalt materials displayed a significant reduction in low-temperature crack resistance.

The reason may be that asphalt materials become harder and more brittle at −24 °C. For one thing, the movement of bitumen molecules is weakened at low temperature; for another thing, the obstruction ability of eggshell powder to asphalt molecules is significantly enhanced, which changes the interaction of asphalt molecules. With the increase of the content, this obstruction ability is stronger, which makes the cracking resistance of asphalt materials at −24 °C more sensitive.

Mechanical response analysis at the above three temperatures demonstrated that ES could not raise the low-temperature crack resistance of the bitumen materials.

## 4. Discussion

This research is intended to provide a new concept for the use of biological waste eggshells. Sustainable construction materials can be produced by applying biological waste to engineering construction. The novelty and scientific contributions of this study are as follows:

- For the sake of solving the environmental and resource issues generated by biological waste eggshells, this study combined eggshell powder with bitumen materials for bio-roads construction, formulated a preliminary set of effective schemes for recycling waste eggshells into asphalt materials, and contributed to the sustainable development of engineering and construction materials.
- This study intended to establish a link between asphalt materials and food bioscience. Moreover, this study will raise important functions for the interdisciplinary study of asphalt materials and food bioscience, and promote the application of biomaterials from food science to engineering.
- Generally, biomass is used as renewable bio-modifiers, including soybean oil, waste engine oil, vegetable oil, palm oil, waste cooking oil, swine manure, and so on. The low-temperature property can be improved by most bio-modifiers, and some bio-modifiers need to adopt pyrolysis, distillation, purification, and other processes, which increases the cost of using biological resources. However, the biological waste eggshell can not only significantly improve asphalt properties inexpensively, but also has a wide range of resources. Therefore, biological waste eggshell is a good biological waste resource, which is worth popularizing and applying.

## 5. Conclusions

This study reveals the high- and low-temperature characteristics of bitumen materials mixed with waste eggshell powder. The following conclusions were obtained:

1. SEM tests showed that eggshell powder has a rough and porous microstructure. This implies that eggshell powder could adsorb more asphalt material and thereby affect the performance of asphalt.
2. The FT-IR test indicated that the eggshell powder could not alter the chemical structure of the bitumen-binding materials, and the eggshell powder and asphalt materials were mainly physical miscibility processes.
3. Asphalt conventional performance tests indicated that ES increased the consistency, hardness, and thermal stability of bitumen materials and reduced the plastic deformation capacity. This is a preliminary indication that eggshell powder can rise the high-temperature characteristics of bitumen.
4. DSR temperature and frequency sweep experiments demonstrated that the eggshell powder could rise the |G*|, reduce the phase angle δ, and improve |G*|/sinδ. This further indicated that eggshell powder could enhance the high-temperature characteristics of bitumen.
5. MSCR revealed that the addition of eggshell powder increased R and reduced J_nr_. This demonstrated that eggshell powder could increase the permanent deformation resistance of asphalt materials.
6. BBR revealed that the addition of eggshell powder could improve the stiffness modulus and reduce the creep rate. This indicated that eggshell powder could not increase the toughness or low-temperature crack resistance of bitumen. The temperature should be considered in practice.

Overall, the addition of waste eggshell powder has an effective influence on the high-temperature characteristics of 70# road bitumen materials. Considering the practice of solid waste management, the environmental policy of reducing solid waste generation and promoting comprehensive utilization, and people’s advocacy of green and low-carbon lifestyles, this sustainable development will greatly improve the environmental and economic impact of the food and construction industries under the condition of increasing demand for asphalt materials in infrastructure construction. This study indicated that the application of waste eggshell powder to asphalt is feasible, promising, and has long-term environmental and resource advantages.

However, this paper aimed to study high- and low-temperature characteristics of bitumen materials. Other characteristics such as the water stability and anti-aging of bitumen materials were not explored. The viscoelastic and rheological characteristics of materials systematically by using the master and black curves were not analyzed. The mechanism of interaction between eggshell powder and asphalt is also unclear. The correlation between eggshell powder and asphalt will be analyzed by atomic force microscope (AFM) experiment. Besides that, the performances at the level of mixture could be studied further, including mixture proportion design, and mechanical property and durability tests. Furthermore, little research was performed on the relationship between eggshell powder and mineral powder, and whether it can replace non-renewable mineral powder resources in the future. Moreover, the properties of eggshell powder bitumen are still lacking practical engineering verification, so test roads will be paved for follow-up observation.

In summary, the future research is recommended to concentrate more on the interaction mechanism between biological waste and asphalt. It is also meaningful to develop a new composite biomaterial that can comprehensively improve the properties of bitumen. More recommendations are as follows:

- Study the viscoelastic and rheological characteristics of materials systematically by using the master and black curves.
- Investigate the relationship between macroscopical performance and the microstructure and chemical composition of asphalt.
- Evaluate the compounding effect of various biological waste asphalt materials.
- Find out the relationship between biological waste and performance of asphalt mixtures.
- Develop a new composite material that can comprehensively improve asphalt performance.
- Pave the test bio-road and observe the performance.

## Figures and Tables

**Table 1:** Applications of municipal waste in construction materials.

Materials	Resources	Application
cement	municipal solid waste incineration fly ash [8]	produce eco-friendly supplementary cementitious materials in the manufacture of eco-cements
	textile waste [9]	produce lightweight concrete partition
	seashell waste [10]	use seashell waste as fine aggregate or cement substitute
	clay brick waste [11]	replace cement with brick waste powder to produce sustainable construction materials
	glass waste [12]	replace cement with ground glass waste
	eggshell ash and strap plastic waste [13]	replace cement with eggshell ash and reinforced with waste plastic fiber
asphalt	fish scale powder [14]	use fish scale powder to improve asphalt performance
	waste wood [15]	use bio-oil extracted from waste wood to rejuvenate asphalt binder
	waste steel shavings and waste ferrites [16]	use waste steel shavings and ferrites to heal pavement crack by induction heating
	plastic waste [17]	improve rutting and fatigue performance asphalt
	dry battery waste powder [18]	improve high-temperature characteristics of asphalt
	swine manure [19]	ameliorate low-temperature characteristics of asphalt
others	electronic wastes (polyethylene) [20]	produce a construction part with electromagnetic- interference-shielding characteristics
	city sewage sludge [21]	produce clay bricks inexpensively
	residue of municipal solid waste pyrolysis [22]	manufacture ceramic material

**Table 2:** Requirements of bitumen.

Properties	Experiment Results	Technical Requirements (JTG F40-2004) [48]
Penetration (25 °C, 100 g, 5 s, 0.1 mm)	66.5	60–80
Softening point (°C)	49.5	≧46
Ductility (5 cm/min, 5 °C, mm)	78	-
Dynamic viscosity (60 °C) (Pa·s)	280	≥180
Flash point (°C)	310	≥260
Wax content (%)	1.81	≤2.2

## Data Availability

The data are not be shared due to restrictions eg privacy and regulation.
